# Multiple anatomic sites of infarction in a pediatric patient with *M. pneumoniae* infection, a case report

**DOI:** 10.1186/s12887-021-02845-3

**Published:** 2021-08-31

**Authors:** Devon W. Hahn, Claire E. Atkinson, Matthew Le

**Affiliations:** 1 Department of Pediatrics, University of Oklahoma Health Sciences Center, 1200 Children’s Avenue, A1 Room 12305, Oklahoma City, Oklahoma 73104 USA; 2 Department of Pediatrics, University of North Carolina, 030 MacNider Hall, CB 7231, Chapel Hill, North Carolina 27599-7231 USA

**Keywords:** mycoplasma, Coagulopathy, Infarction, Embolism, Endocarditis, Pediatrics

## Abstract

**Background:**

Although *M. pneumoniae (M. pneumoniae)* infections have been associated with various extrapulmonary manifestations, there have been very few documented cases of thrombotic events in pediatrics, and none to our knowledge with such extensive involvement as the patient described here. We aim to contribute to the urgency of discovering the mechanism of the coagulopathy associated with *M. pneumoniae* infections.

**Case presentation:**

This 10-year-old boy was admitted after 2 weeks of fever, sore throat, worsening cough, and progressive neck and back pain. During hospitalization, he developed clots in several different organs: bilateral pulmonary emboli, cardiac vegetations, multiple splenic infarcts, and deep venous thromboses in three of four extremities. He was treated with long-term antibiotics and anticoagulation, and fully recovered.

**Conclusions:**

This is the first case known to us of a child with an extensive number of thrombotic events in multiple anatomic sites associated with *M. pneumoniae* infection. The mechanism by which *M. pneumoniae* infection is related to thrombotic events is not fully understood, but there is evidence that the interplay between the coagulation pathways and the complement cascade may be significant. This patient underwent extensive investigation, and was found to have significant coagulopathy, but minimal complement abnormalities. By better understanding the mechanisms involved in complications of *M. pneumoniae* infection, the clinician can more effectively investigate the progression of this disease saving time, money, morbidity, and mortality.

## Background

*M. pneumoniae(M. pneumoniae)* is a common infection in children, responsible for up to 40% or more of cases of community-acquired pneumonias and as many as 18% of cases requiring hospitalization in children [1]. Up to 25% of these patients experience extrapulmonary complications. The most commonly recognized extrapulmonary complication is central nervous system (CNS) involvement, and ranges from encephalitis to cranial nerve palsies to acute disseminated encephalomyelitis [1]. Dermatologic manifestations are also common, and occur in up to 25% of patients. Cardiac complications are uncommon, but have involved pericarditis, myocarditis, endocarditis, and cardiac tamponade, and may be related to direct detection of the organism in cardiac tissue or involve an autoimmune mechanism [2]. Septic arthritis, rhabdomyolysis, an assortment of non-specific gastrointestinal symptoms, and renal complications (including acute glomerulonephritis, interstitial nephritis, nephrotic syndrome, and IgA nephropathy presumable as a result of immune complex formation), have all been identified [2].

.Although *M. pneumoniae* infections have been associated with various extrapulmonary manifestations, there have been very few documented cases of thrombotic events in pediatrics, and none to our knowledge with such extensive involvement as the patient described here. We aim to contribute to the urgency of discovering the mechanism of the coagulopathy associated with *M. pneumoniae* infections.

## Case presentation

A previously healthy 10-year-old male was admitted after 2 weeks of fever, sore throat, non-productive cough, progressive difficulty breathing, and worsening neck and back pain. Vital signs on admission revealed a fever of 38.1 C, pulse of 139 beats per minute, respiratory rate of 36, and a blood pressure of 140/76 mm Hg (mm/Hg), but his oxygen saturations were 100% on room air. Inflammatory markers were elevated: C-reactive protein (CRP) 146 mg/Liter (mg/L), procalcitonin 25 nanograms/milliliter (ng/mL), and lactate dehydrogenase (LDH) 1200 units/Liter. A chest x-ray and chest computerized tomography (CT) showed a left upper lobe consolidation, and he was admitted to the hospital for severe sepsis and pneumonia. He was started on vancomycin and ceftriaxone.

On hospital day 3, he complained of increasing right-sided neck pain. Exam showed a febrile, ill-appearing boy, shivering, and fearful during exam, but he was alert and oriented. The right side of his neck was significantly tender with limited range of motion. He had significant diffuse non-tender scalp edema and mild edema of hands and feet as well, but did not complain of any pain in those areas. Chest exam revealed significantly diminished breath sounds over the left hemithorax, but no crackles or wheezing. He had nasal flaring while sleeping, and oxygen saturations of > 94% on 2 l/minute of oxygen by nasal canula while both awake and asleep. Labs at that time showed a normal white blood cell count of 13.42 thousand/cubic millimeter (K/mm^3^), normal platelet count of 180 K/mm^3^and a hemoglobin of 8.9 g/deciliter (g/dL). Red blood cell indices showed a mean corpuscular volume (MCV) of 86 femtoliters (fL, or 10^− 15^ l), a mean corpuscular hemoglobin (MCH) of 26 picograms (pg), and a mean corpuscular hemoglobin concentration (MCHC) of 35.6 g/dL. Coagulation studies were elevated, with a prothrombin time of 26.1 s, International Normalized Ratio (INR) of 2.3, and an activated partial thromboplastin time (aPTT) of 42.3 s; however, fibrinogen was normal at 311 mg/deciliter (mg/dL). D-dimer was dramatically elevated to 17,679 nanograms/milliliter D-dimer units (ng/ml DDU). Lactate dehydrogenase (LDH) was elevated to 1249 units/Liter, and uric acid was low at 1.8 mg/dL. Total protein was low normal at 6.0 g/dL, and albumin was low at 2.8 g/dL. Vitamin K was given prophylactically for increased risk of bleeding due to coagulation abnormalities.

**Fig. 1:**
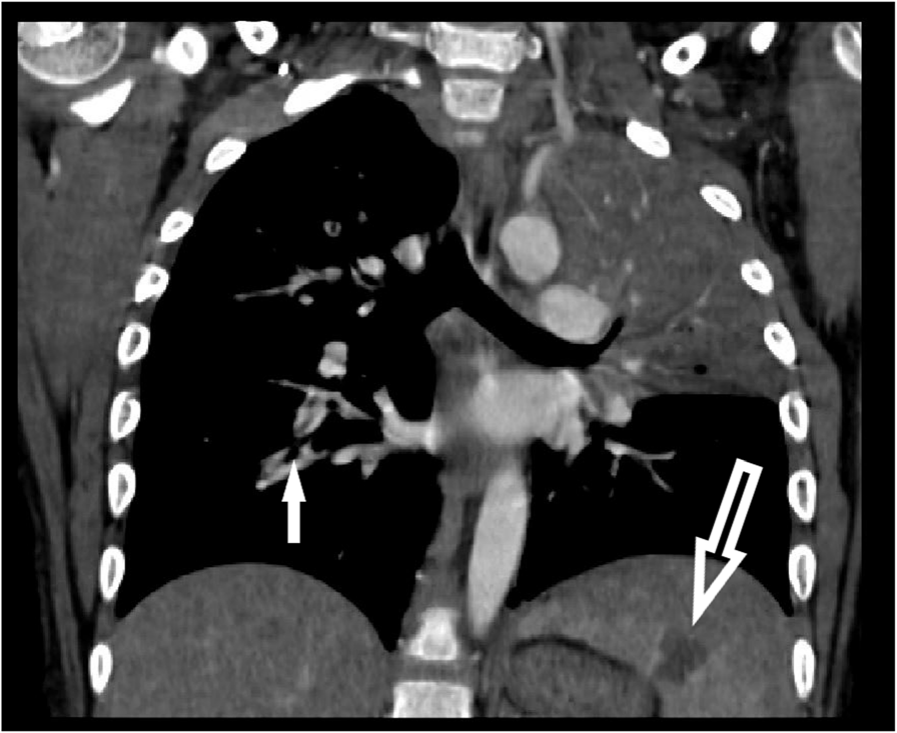
CT of chest, coronal view, showing consolidation in Left upper lobe of lung, large splenic infarct (large open arrow), and filling defect of a Right pulmonary embolus (small solid arrow)

CT of the chest showed complete consolidation of the left upper lobe, bilateral pulmonary emboli, as well as multiple wedge-shaped splenic infarcts. (Fig. 1, below). CT of the neck showed a non-occlusive venous thrombus in a right-sided internal jugular tributary vein. Head CT was normal. Trans-thoracic echocardiogram was read as normal without vegetations, but a trans-esophageal echocardiogram (TEE) showed a small echogenic slightly mobile mass along right side of the ventricular septum. Doppler ultrasound of the extremities showed multiple deep venous thromboses in bilateral lower and right upper extremities, (which may have accounted for the edema in the distal extremities on exam). He was transferred to the Intensive Care Unit for initial heparinization with 35 units/kilogram (U/kg) over 10 min loading dose, then a 20 U/kg intravenous drip to achieve anti-Xa levels between 0.3–0.7 international units/milliliter (IU/mL), then transferred back to the floor for continued low molecular weight heparin with enoxaparin (Lovenox) to maintain therapeutic anti-Xa levels between 0.5–1.0 IU/mL. One of two blood cultures from the referring facility became positive for *Rothia dentocariosa (R. dentocariosa)*, and was initially believed to be a contaminant, since the second blood culture drawn at the same time remained negative. Upon further questioning, it was discovered that this patient had a remote history of dental surgery, but none in the recent past. However, because of the mobile mass seen on TEE, the consensus was to manage as presumed bacterial endocarditis with long-term antibiotics. Sensitivities performed on the identified *R. dentocariosa* showed sensitivity to vancomycin with a minimum inhibitory concentration (MIC) of <=1 microgram/milliliter (mcg/mL), and was found to be intermediately sensitive to penicillin with an MIC of 0.5 mcg/mL.

On hospital day 8, his hemoglobin fell to 5.9 g/dL, requiring a red blood cell transfusion. It was believed to be a non-hemolytic anemia, as haptoglobin was elevated to 209 mg/dL, LDH had normalized at 471 units/liter, and uric acid was low at the time at 2.1 mg/dL. A thorough workup for clotting deficiencies was begun. All values were normal except for von Willebrand Factor and Cofactor (see Table 1 below) which were both elevated; reasons for these factors being elevated are unclear. A bone marrow aspirate and biopsy showed trilineage hematopoiesis, no pathologic cells, and were negative for malignancy.

**Table 1:** Lab Results (Bolded results are abnormal)

**Clotting Factors**	Result	Reference Range
Factor VIII	146%	50–150%
Von Willebrand Factor antigen	> 200%	60–150%
Von Willebrand Ristocetin Cofactor	> 200%	50–150%
Lupus Anticoagulant Sensitive PTT	51.5 s, corrects with mixing	28–47 s; No evidence of Lupus
dRVVTS (Dil Russell Viper Venom Time Screen)	70.3 s, corrects with mixing	33–61 s
Factor V	121%	50–150%
Factor VII	75%	50–150%
Factor IX	98%	50–150%
Protein C Activity	76%	70–150%
Protein S free antigen	76%	65–150%
Functional Anti-thrombin III	94%	83–128%
Factor V Leiden mutation	Not detected	
PTT mixing study with heparinase	45.5 s	
**Immunology**	Result	Reference range
Total Immunoglobulin (Ig) IgG	**2082 mg/dL**	698–1560
IgG1	**1340 mg/dL**	309–813
IgG2	**413 mg/dL**	94–389
IgG3	**193 mg/dL**	20–100
IgG4	32 mg/dL	4–110
IgA	**377 mg/dL**	81–233
IgM	**425 mg/dL**	42–143
Antinuclear antibodies (ANA) Screen	**Positive (HEp-2 test)**	Neg or < 40
ANA Titer	**40**	< 40
ANA Pattern	**Nuclear Speckled Immunofluorescence Assay (IFA)**	
ANA Pattern 2	**Cytolplasmic IFA**	
Anti-beta-2-glycoprotein antibodies IgG	< 2 G units	< 20
Anti-beta-2-glycoprotein antibodies IgM	< 2 M units	< 20
Anti-cardiolipin IgG	< 2 IgG Phospholipid units (GPL)	0–23
Anti-cardiolipin IgM	< 2 GPL	0–23
Total Complement (CH50)	Normal	
Complement C3	110 mg/dL	83–157
Complement C4	**8.2 mg/dL**	13–35
Total Complement (CH50)	51 Units/mL	40–99,999
Total Lymphocytes	3597 cells/microliter	
Absolute CD3 Count	3273 cells/microliter	800–3500
Absolute CD4 Count	**2482 cells/microliter**	400–1200
*M. pneumoniae* IgG	**2261 Units/ml**	0–99
*M. pneumoniae* IgM	**5882 Units/ml**	0–769
Anti-streptolysin O	**810 Units/ml**	< 250
*Aspergillus* Antigen	0.08 Index	0.00–0.49
Beta-(1,3)-D-Glucan	< 31 picograms/mL	< 80
*B. henselae* IgG titer	Negative	Negative = < 1:320
*B. henselae* IgM titer	Negative	Negative = < 1:100
*B. quintana* IgG titer	Negative	Negative = < 1:320
*B. quintana* IgM titer	Negative	Negative = < 1:100
Urine *Histoplasma* antigen	None detected	
HIV 1&2 antibody	Nonreactive	
TB Quantiferon	Negative	Negative
**Microbiology**	Result	
Blood cultures on Hospital Days 2, 3, and 4	Negative	
Blood culture Hospital Day 6	***Rothia dentocariosa***	
Blood cultures Hospital Days 9 and 11	Negative	
Bone Marrow Biopsy Cytomegalovirus	Negative	
Bone Marrow Biopsy AFB	Negative	
Bone Marrow Biopsy GMS	Negative for fungal elements	
Bronchoalveolar Lavage (BAL) viral culture	Negative	
BAL *Mycoplasma pneumoniae* culture	Negative	
BAL Cytomegalovirus (CMV) Shell Vial culture	Negative	
BAL *C. trachomatis* culture	Negative	
BAL Acid Fast Bacilli culture	Negative	
BAL Calcofluor Prep	Negative for fungal elements	
BAL anaerobic culture	Negative	
BAL Pneumocystis DFA	Negative	
BAL CMV Shell vial isolate	Negative	

Inflammatory markers continued to be elevated throughout his hospitalization: CRP peaked at 146.6 mg/L, erythrocyte sedimentation rate (ESR) was > 145 mm/hour (mm/hr) and ferritin was 704 ng/ml. Immunoglobulin (Ig) levels were found to be high and included total IgG, IgG subclasses, IgA, IgM, and *M. pneumoniae* specific IgG and IgM. Further investigation revealed the presence of red blood cell agglutination (cold agglutination testing was not performed) and positive lupus anti-nuclear antibodies with nuclear speckled and cytoplasmic patterns. All other immune workup was negative, including anti-Phospholipid antibodies (anti-beta-2 glycoproteins IgG and IgM, and anti-cardiolipin IgG and IgM) (see Table 1 below).

The problem list was expanded to include *Mycoplasma pneumonia* infection, bacteremia with *Rothia dentocariosa*, infective endocarditis from *M. pneumoniae* and/or *R. dentocariosa*, coagulopathy, bilateral pulmonary embolism, deep vein thrombosis of bilateral lower extremities and right upper extremity, multiple splenic infarcts, and a right internal jugular tributary vein thrombosis.

This patient received a 10-day course of azithromycin for the *M. pneumoniae* infection. For treatment of presumed infective endocarditis with *R. dentocariosa*, he was also continued on vancomycin through hospital day 9, at which time antibiotics were changed to ampicillin/sulbactam. The ampicillin/sulbactam was continued after discharge for 8 weeks, when the ESR and CRP had returned to normal and echocardiogram showed resolution of the vegetation. He was continued on home Lovenox until echocardiogram, CT of chest, and bilateral lower extremity vascular ultrasound all showed resolution of thromboses, a total of 5 months.

On follow up, the patient is doing well. He has had no untoward sequelae. He, his mom, and grandmother describe him as “back to normal,” bored because of the pandemic, but otherwise well.

## Discussion and conclusions

This case describes a patient with presumed *M. pneumoniae* infection. The infectious etiology is confounded by a single blood culture positive for *Rothia dentocariosa*. Either of these organisms could have been responsible for the presumed sepsis and endocarditis, and both infections were fully treated. While infective endocarditis and sepsis can certainly cause thromboembolic events, the added presence of *M. pneumoniae* begs the question of this organism’s contribution to the vast extent of clotting seen in this patient.

There are very few cases published that involve thrombotic events associated with *M. pneumoniae* infection. Flateau et al. described a review of the literature that identified 23 patients (18 were children) with extrapulmonary thrombotic manifestations [3]. There are a few other case reports of thrombosis and infarction; some examples follow: Park et al. described a 12 year old child who died of multi-organ involvement, including myocardial infarction, in association with mycoplasma infection [4]. Choi et al. described a 5 year old boy who had an extensive stroke in multiple cerebrovascular territories 10 days after the diagnosis of *M. pneumoniae* infection [5]. And a 12 year old was described as having priapism, most likely due to the hypercoagulable state induced by concomitant mycoplasma infection [6]. However, there are no reports to date that describe the extensive thromboses seen in our patient. Fortunately, this patient had no CNS lesions, and recovered fully.

Although the mechanism of hypercoagulability in *M. pneumoniae* infections is unknown, several investigators have proposed different theories. It has been proposed that *M. pneumoniae* may act more like a virus than a bacterium when invading a host organism, and the host’s immune responses may contribute to the pathogenesis of disease. Substances produced from pathogens, including toxins and pathogen-associated molecular patterns, as well as those produced by damaged infected host cells such as damage-associated molecular patterns, pathogenic proteins, and pathogenic peptides all contribute to the inflammatory response of the host. In Kyung-Yil Lee’s proposed Protein-Homeostasis-System hypothesis, both the pathogen and the host response are responsible for disease. “Every infectious disease has its own set of toxic substances, including pathogenic proteins, associated with disease onset, and the pathogenic proteins and the corresponding immune cells may be responsible for the inflammatory processes that develop in those infectious diseases.” [7] The disruption of this homeostasis produces an intense inflammatory response, and causes damage and destruction in areas unrelated to the original site of infection, which could account for the wide range of extrapulmonary manifestations seen in some *M. pneumoniae* infections.

Mitsuo Narita published three possible mechanisms at work in the manifestation of extrapulmonary disease in mycoplasma infection: 1) a direct type in which bacterium is present at the site of inflammation and local inflammatory cytokines induced by the bacterium play an important role, (2) an indirect type in which the bacterium is not present at the site of inflammation and immune modulations, such as autoimmunity or formation of immune complexes, play an important role, and (3) a vascular occlusion type in which obstruction of blood flow induced either directly or indirectly by the bacterium plays an important role [8].

.Flateau et al. described how immune mediation may also play a part in the hypercoagulability seen in *M. pneumoniae* infection: “Antiphospholipid antibodies (aPL), including anticardiolipin antibodies (aCL) and lupus anticoagulant, react against proteins that bind to phospholipids on plasma membranes, contributing to thrombosis. The presence of transient aPL associated with viral, bacterial and parasitic infections is well documented, but their pathogenic role is uncertain. However, in *M. pneumoniae* infection, several cases of transient aPL associated with thrombosis have been reported, suggesting that they might actively contribute to hypercoagulability.” [3].

Chapin et al. describes an association between the complement system and the coagulation cascade, as interactions between the two have been well characterized by in vitro studies. Complement pathways, especially the alternative complement pathway, are activated in hemolytic anemias and are closely linked with thrombosis [9]. Multiple coagulation proteases exhibit complements C3 and C5 convertase formation, including thrombin, coagulation factors Xa and XIa, and plasmin [10–12]. However, it is still unclear how the interaction of these two systems impacts the development of thrombosis.

It has long been established that *M. pneumoniae* activates agglutination of red blood cells in cold environments. Cold agglutinin mediated hemolytic anemia is a well-documented phenomenon. The cold agglutination effects of *M. pneumoniae* may also be indirectly responsible for coagulopathy and thrombosis. The association between cold agglutination and the activation of the complement cascade has been previously described [10], but the role that the coagulation cascade plays in the development of thrombotic events is not well understood.

The authors admit there are limitations to the confirmation of diagnosis of *M. pneumoniae* in this case. Many studies have compared different kits available and assay methods used for the diagnosis of *M. pneumonia* [13–15]. It is recommended that paired specific IgM and IgG serologic tests two to 4 weeks apart are needed to confirm *M. pneumoniae* [14, 16, 17]. This facility utilized an EIA assay method by GenBio for the serologic evaluation of *M. pneumoniae* using IgM and IgG levels, and in the case presented here a single evaluation of IgM and IgG levels was performed. Both IgM and IgG were high in this case, however the PCR was negative for *M. pneumoniae*. In most studies comparing assay methods from the different kits available, the serology is more likely to be negative early in the disease process, and shows high levels of IgM and IgG 2–4 weeks into the infection course. Since this patient had been ill for 2 weeks prior to IgM and IgG levels being drawn, it is likely that these levels reflect current infection with *M. pneumoniae*. However, it is possible that the positive results in this case are reflective of previous infection, as immunity and positive testing may last as long as 1–4 years after infection [18].

.The patient presented here had no antiphospholipid antibodies, and had very weakly positive anti-nuclear antibodies. The significance of this is not clear. Complement testing showed no abnormalities, except for the decrease in C4 (see Table 1 below). Furthermore, while red cell agglutination was demonstrated and the presenting anemia may have been caused by a hemolytic process, the severe drop in hemoglobin on day 8 did not appear to be due to hemolysis, as LDH was normal and uric acid was low at that time. So, while there are theories about the process of the coagulopathy associated with *M. pneumoniae* infection, this case illustrates the complexity of determining the exact mechanisms involved.

This is a rare case of a child with multiple sites of thromboses in the setting of *M. pneumoniae* infection. Although the well-known association of red cell agglutination was demonstrated in this case, it was not responsible for the sudden drop in hemoglobin after 3 weeks of illness, nor does it explain the increased coagulability and multiple sites of thromboses. There were increased levels of anti-nuclear antibodies, but it is unclear if that contributed to the hypercoagulable state. Because the relationship between complement and coagulation is not well understood as of yet, the normal values for complement in this patient cannot be interpreted. It is evident from this case that more research is needed to accurately describe the phenomenon of coagulopathy and thrombosis in *M. pneumoniae* infections.

## Data Availability

Data sharing is not applicable to this article as no datasets were generated or analyzed during the current study. The data provided in this case report was obtained from the electronic medical records of Oklahoma Children’s Hospital and is protected health information. Consent for sharing this de-identified information was obtained from the parent of the patient, and is available upon request.

## Abbreviations

*M. pneumoniae*: *Mycoplasma pneumoniae*
mm/Hg: Millimeters of mercury
CRP: C-reactive protein
ng/mL: Nanograms/milliliter
LDH: Lactate dehydrogenase
CT: Computerized tomography
K/mm^3^: Thousand/cubic millimeter
g/dL: Grams/deciliter
MCV: Mean corpuscular volume
fL, or 10^− 15^ l: Femtoliters
MCH: Mean corpuscular hemoglobin
pg: Picograms
MCHC: Mean corpuscular hemoglobin concentration
INR: International Normalized Ratio
aPTT: Activated partial thromboplastin time
mg/dL: Milligrams/deciliter
ng/ml DDU: Nanograms/milliliter D-dimer units
TEE: Trans-esophageal echocardiogram
U/kg: Units/kilogram
IU/mL: International units/milliliter
*R. dentocariosa*: *Rothia dentocariosa*
MIC: Minimum inhibitory concentration
mcg/mL: Microgram/milliliter
ESR: Erythrocyte sedimentation rate
Ig: Immunoglobulin
CNS: Central nervous system
ANA: Antinuclear antibodies
aPL: Antiphospholipid antibodies
aCL: Anticardiolipin antibodies
BAL: Bronchoalveolar lavage
CMV: Cytomegalovirus
dRVVTS: Dil Russell Viper Venom Time Screen
IFA: Immunofluorescence Assay
GPL: IgG Phospholipid units
